# Enabling scalable optical computing in synthetic frequency dimension using integrated cavity acousto-optics

**DOI:** 10.1038/s41467-022-33132-z

**Published:** 2022-09-15

**Authors:** Han Zhao, Bingzhao Li, Huan Li, Mo Li

**Affiliations:** 1 Department of Electrical and Computer Engineering, University of Washington, Seattle, WA 98195 USA; 2 Department of Physics, University of Washington, Seattle, WA 98195 USA

**Keywords:** Microwave photonics, Integrated optics, Nanocavities, Silicon photonics

## Abstract

Optical computing with integrated photonics brings a pivotal paradigm shift to data-intensive computing technologies. However, the scaling of on-chip photonic architectures using spatially distributed schemes faces the challenge imposed by the fundamental limit of integration density. Synthetic dimensions of light offer the opportunity to extend the length of operand vectors within a single photonic component. Here, we show that large-scale, complex-valued matrix-vector multiplications on synthetic frequency lattices can be performed using an ultra-efficient, silicon-based nanophotonic cavity acousto-optic modulator. By harnessing the resonantly enhanced strong electro-optomechanical coupling, we achieve, in a single such modulator, the full-range phase-coherent frequency conversions across the entire synthetic lattice, which constitute a fully connected linear computing layer. Our demonstrations open up the route toward the experimental realizations of frequency-domain integrated optical computing systems simultaneously featuring very large-scale data processing and small device footprints.

## Abstract (web summary)

Synthetic frequency dimension from light modulation enables scalable optical computing. The authors show an efficient silicon-based acousto-optic modulator that generates large synthetic frequency lattices and performs matrix-vector multiplications.

## Introduction

Analog optical computing encodes and processes data using continuously variable quantities of light^1–3^. While optical nonlinearity requires high power expense, linear optical components can perform data movement, temporal-spatial signal processing and multiply-accumulate operations with potentially unparalleled bandwidth, speed and energy efficiency^4–7^. As the current digital electronic computing technologies approach the physical limit, such advantages of optics motivate the recent development in building optical accelerators that can sustain the ever-growing data demand at the hardware level^8–16^. Integrated photonics provides a powerful optical computing platform that benefits from scalable fabrications and integration compatibility with electronic circuits, affording architectures with rapid programmability^11–16^. Considerable progress has been made in building integrated photonic neural networks with high data throughput by incorporating time and/or wavelength division multiplexing^15,16^. However, realizing large-scale, fully connected networks on photonic chips can be very challenging. Most *N* × *N* optical computing layers based on spatial encoding require *O*(*N*^2^) scaling of photonic components, occupying huge device footprints compared to the electronic counterparts. Such footprint-inefficient scaling poses as one of the roadblocks for integrated photonic computing from being applied in some important architectures^17^, which will require efforts in multiple technological areas to resolve.

The emerging notion of synthetic frequency dimension provides a promising strategy to drastically scale up the optical computing systems in both classical and quantum regimes^18–25^. Encoding information as coherent optical fields on a synthetic frequency lattice increases the fan-in/fan-out of a single photonic logic unit, thus improving the scalability of data processing by orders of magnitudes. The implementations of frequency-domain *N* × *N* optical networks require efficient modulators that simultaneously link the *N* discrete nodes via coherent frequency conversions^19,25^. For this purpose, while the electro-optic modulators (EOMs) provide broadband modulations^26,27^, integrated acousto-optic modulators can stand out with high modulation efficiency and large modulation depth with much reduced device footprint by exploiting the strong optomechanical interaction between co-localized optical and acoustic modes^28–33^. Recent thin-film lithium niobate modulators have reached modulation depth that can couple a few sidebands^31,33^. Nonetheless, a single device that can compose a fully connected computing layer on a sizable synthetic frequency lattice remains unrealized. Achieving the most efficient on-chip acousto-optic modulation requires simultaneously optimized optomechanical coupling and piezoelectric transduction on a monolithic material platform. To this end, the heterogeneous integration of silicon on insulator (SOI) with complementary metal-oxide-semiconductor (CMOS)-compatible piezoelectric materials such as aluminum nitride (AlN) holds promise for high-performance large-scale integrated modulators^34–36^, which will offer the key building blocks for data-intensive frequency-domain optical computing systems.

**Fig. 1:**
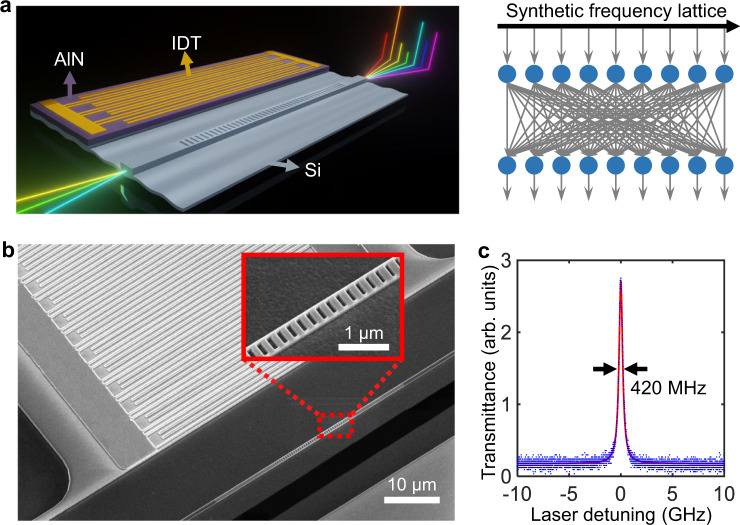
Nanophotonic cavity acousto-optic modulator that performs scalable matrix-vector multiplications in the synthetic frequency dimension. **a** Schematic illustration of our acousto-optic modulator, which is equivalent to a fully connected linear optical computing layer on the synthetic frequency lattice. **b** SEM image of the modulator fabricated on the AlN-on-SOI platform. Inset is the zoom-in image of the nanophotonic cavity. **c** Measured optical transmission spectrum without modulation, showing a linewidth of 420 MHz. The center optical resonance wavelength is around 1547.5 nm.

Here, we demonstrate scalable matrix-vector multiplications (MVM))—an essential computation step in algorithms such as neural networks—in the synthetic frequency dimension by leveraging an efficient nanophotonic cavity acousto-optic modulator on the AlN-on-SOI platform. The very large dynamic modulation depth arising from the engineered strong electro-optomechanical coupling enables the coherent frequency conversions among a myriad of sidebands spanning a synthetic frequency lattice. Thereby, with a single such modulator, we realize a large-scale, fully connected computing layer that performs linear transformations on the complex-valued vector inputs encoded as spectrally coherent optical fields (Fig. 1a). We highlight the advantage of the persistent long-range spectral phase coherence of the MVM operations performed by our modulator. Our device does not realize a full optical computing system, but contributes the critical component to a highly scalable and hardware-efficient integrated photonic computing architecture based on cascaded layers of modulators.

## Results

### Design and characterizations of the acousto-optic modulator

**Fig. 2:**
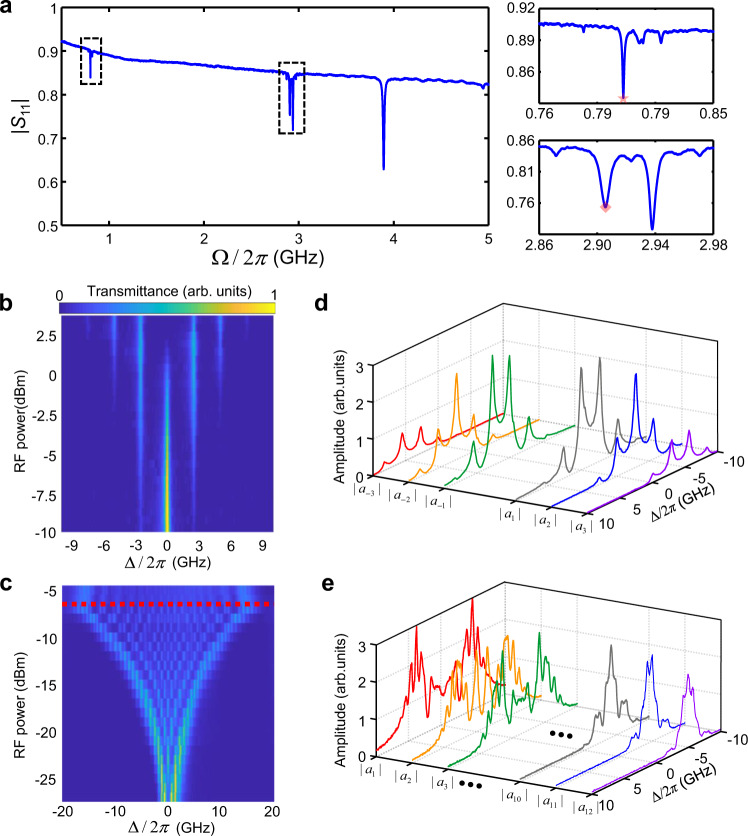
Characterizations of the acousto-optic modulation efficiency and the harmonic signal generations. **a** Reflection (amplitude) spectrum of the IDT. The mechanical resonances in the zoom-in spectra at ~800 MHz and ~2.90 GHz have pronounced modulation efficiency, whereas the resonance at 3.95 GHz has a negligibly weak modulation effect. The star and diamond marks denote the prominent frequencies (803 MHz and 2.903 GHz) with enhanced modulation efficiencies for the corresponding mechanical modes (see Methods). **b** Optical transmission spectra under the modulation driven at 2.903 GHz with varying RF power. **c** Optical transmission spectra under the modulation driven at 803 MHz with RF power. The red dashed line denotes the threshold of an electromechanical nonlinearity above which the number of sidebands ceases increasing. **d** Measured optical spectra of the non-vanishing harmonic signal amplitudes |*a*_*l*_|(*l* = ±1, ±2, ±3) at 2.903 GHz, −2.5 dBm RF drive (*β* = 1.29). **e** Spectra of the positive-order harmonic signal amplitudes |*a*_*l*_|(*l* = 1, 2, 3, …, 10, 11, 12) at 803 MHz, −17 dBm RF drive (*β* = 6.90). The higher-order harmonics decrease significantly beyond *l* = 12 at this drive. The corresponding spectra of the negative-order harmonics (not plotted) are mirror-symmetric about Δ = 0. Colors of the curves in (**d**) and (**e**) denote the corresponding harmonic orders.

Figure 1b shows a scanning electron microscopy (SEM) image of our device fabricated on an AlN-on-SOI substrate (see Methods and Supplementary Note 1). The modulator consists of a one-dimensional photonic crystal cavity etched in a suspended silicon rib waveguide which is connected to the AlN/Si piezoelectric region with a silicon sleeve area in between. The nanophotonic cavity is end-coupled to a pair of grating couplers for optical input/output. We achieved a high loaded quality factor *Q*_L_ = 4.6 × 10^5^, corresponding to a total cavity loss rate of *κ* = (2π) ∙ 420 MHz (Fig. 1c). Acoustic waves are launched by driving a split-finger interdigital transducer (IDT) patterned on the free-standing AlN/Si region with an RF signal, and subsequently propagate to the optical waveguide via the silicon membrane. The IDT is designed with a period of 3 μm to excite a set of mechanical modes with angular frequencies Ω > *κ*, reaching the sideband-resolved regime (Fig. 2a) (see Supplementary Note 2). By etching a free-edge reflector on the lower side of the waveguide, we create an acoustic resonator that coherently builds up a strong mechanical displacement field at the nanophotonic cavity. Such mechanical motion effectively modulates the optical resonance through a combination of moving-boundary and photoelastic effects^37^. Under the modulation, the intra-cavity photon dynamics can be described as

1 $$\dot{a}(t)=[i(\varDelta -\beta \cdot \varOmega \cdot \hat{f}(t))-\kappa /2]a(t)+\sqrt{{\kappa }_{{{{{{\rm{ex}}}}}}}}{a}_{{{{{{\rm{in}}}}}}}(t),$$

where *κ*_ex_ is the external coupling rate, *a*_in_(*t*) is the input optical field, and Δ = *ω*_p_ – *ω*_0_ is the detuning of the input laser (angular) frequency *ω*_p_ from the cavity center frequency *ω*_0_. $\hat{f}(t)$ denotes the normalized modulation waveform. *β* = 2*g*_om_/Ω is the modulation index that measures the dynamic modulation depth, where *g*_om_ is the optomechanical coupling proportional to the amplitude of the mechanical mode.

Our acousto-optic modulator features ultra-high modulation efficiency in the sideband-resolved regime. The resulting deep modulation can generate multiple resolved sidebands at *ω*_0_ ± *s*Ω with integer sideband order *s*, forming a synthetic lattice in the frequency domain. To quantify the modulation efficiency and the achievable size of the synthetic lattice, we drive the IDT at the mechanical resonances and measure the optical transmission spectra with varying RF power (see Methods). Figure 2b shows the results of the 2.903 GHz drive (Ω/*κ* ~ 7), from which we infer a characteristic half-wave voltage *V*_π_ = 580 mV for *β* = π by fitting the measurements with the theory (see Supplementary Note 7). At an RF power of 3 dBm (*β* = 2.41), we observe the emergence of multiple sidebands up to ±3rd orders, comprising a finite lattice of 7 sites. The 803 MHz drive (Ω/*κ* ~ 2) can induce much more efficient modulation with the lowest *V*_π_ = 19 mV (Fig. 2c). We obtain the maximal *β* = 22.9 at an RF power of −7 dBm, which generates a synthetic lattice of ~50 sites over a wide frequency range of 40 GHz.

### Coherent frequency conversions in the synthetic frequency dimension

The centerpiece of performing fully connected MVM with our modulator is the coherent conversions from each input frequency site to all the sites at the output. To understand this, we consider a monochromatic laser input and an RF drive with a single microwave tone $\hat{f}(t)=\,\cos (\varOmega t+\varphi )$. At a large *β*, the incident photons can absorb or emit multiple phonons because of the strong optomechanical coupling. Consequently, the input optical field is scattered to a set of harmonic signals {*a*_*l*_} detuned from the cavity center frequency by Δ + *l*Ω, where *l* is the harmonic order. By solving Eq. (1), it can be derived that

2 $${a}_{l}={\sum }_{k}\,{J}_{l+k}(\beta ){J}_{k}(\beta ){e}^{-il\varphi }\frac{{\kappa }_{{{{{{\rm{ex}}}}}}}{a}_{{{{{{\rm{in}}}}}}}}{i(-\varDelta+k\varOmega )+\kappa /2},$$

where ${J}_{\nu }(x)$ is the *v*-th order Bessel function of the first kind (see Supplementary Note 3). We perform heterodyne measurements to characterize the amplitudes of all the harmonic signals with varying input laser frequency. Figure 2d and Fig. 2e show the exemplary optical spectra of the harmonics measured at the RF drives of 2.903 GHz and 803 MHz, respectively, highly consistent with the theoretical values (see Methods and Supplementary Note 8). When the input laser frequency is set on the *n*-th synthetic lattice sites, i.e., detuned by Δ = *n*Ω, each *a*_*l*_ leads to a non-local frequency conversion from the *n*-th site to the *m*-th site, where *m* = *n* + *l*. Hence, the entire set of harmonic generations at all sidebands constitute a two-dimensional optomechanical coupling tensor

3 $${{{{{\bf{G}}}}}}=[{g}_{mn}],\,m,n\in [-M,M]:\,{g}_{mn}=\frac{{a}_{m-n}(\varDelta=n\varOmega )}{{a}_{{{{{{\rm{in}}}}}}}},$$

where 2 *M* + 1 is the size of the synthetic lattice determined by the modulation index *β*. Each *g*_*mn*_ describes the connection between frequency lattice *m* and *n*. Therefore, tensor **G** represents the fully connected layer realized by our AOM. Since the coupling term *g*_*mn*_ is only non-trivial for input frequency near the cavity resonance and for finite spans of frequency conversions, the size of the effective vector space in the synthetic frequency dimension is always bounded (see Supplementary Note 4). More generally, for an optical input vector on the synthetic lattice ${{{{{\bf{x}}}}}}={({x}_{-M},{{{{\mathrm{..}}}}}.,{x}_{0},{{{{\mathrm{..}}}}}.,{x}_{M})}^{{{{{{\rm{T}}}}}}}$, our modulator performs a complex-valued MVM ${{{{{\bf{y}}}}}}={{{{{\bf{G}}}}}}\cdot {{{{{\bf{x}}}}}}$, yielding an output vector ${{{{{\bf{y}}}}}}={({y}_{-M},{{{{\mathrm{..}}}}}.{y}_{0},{{{{\mathrm{..}}}}}.,{y}_{M})}^{{{{{{\rm{T}}}}}}}$. We highlight that, with *β*_max_ = 22.9, a single such modulator provides an MVM unit with a scalable size of up to 50×50 in the frequency domain.

### Large-scale coherent MVM operations

In addition to the high scalability, another outstanding advantage of the complex-valued MVM in the synthetic frequency dimension is the persistent phase coherence across the entire synthetic lattice. In contrast to conventional spatial-domain schemes, which are susceptible to various causes of computational errors such as device defects, non-uniformity and thermal fluctuations, the phase information transmitted through the synthetic lattice is intrinsically preserved by the coherent photon-phonon interactions in our modulator. To demonstrate the scalable and complex-valued MVM, we operate our device using the Ω = 803 MHz drive and set *β* = 11.3, which generates a 25 × 25 matrix **G** according to Eq. (3). A Mach-Zehnder intensity modulator **M**_**I**_ is used to synthesize a vector input of three coherent frequency components, including the carrier transmission and the two opposite-sign sideband signals with their complex amplitudes controlled by a DC bias and an RF drive at Ω, respectively. For simplicity, we tune the temporal delay of the modulations to be zero (*ϕ* = 0) (see Supplementary Note 9). The output on the synthetic frequency lattice is thereby a result of the weighted complex-number summation of the corresponding columns in **G**, representing the complex-valued MVM operation.

**Fig. 3:**
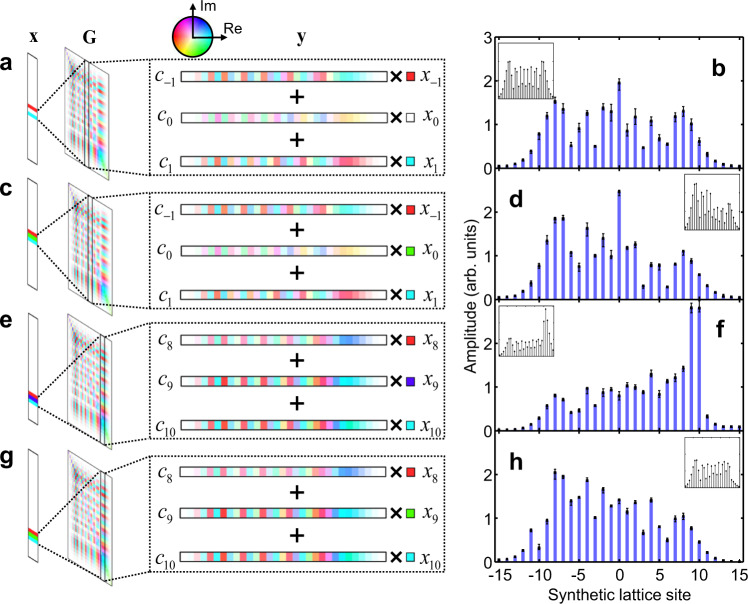
Large-scale coherent matrix-vector multiplications in the synthetic frequency dimension. The large matrix **G** is configured by driving our acousto-optic modulator at 803 MHz with −13 dBm power (*β* = 11.3). The vector input **x** of three coherent frequency components is synthesized and controlled by an intensity modulator **M**_**I**_. **a** MVM with laser frequency at the 0th synthetic lattice site (Δ = 0) and **M**_**I**_ set at suppressed carrier transmission. **c** Δ = 0 and **M**_**I**_ set with three equal-amplitude components and a negative phase on the carrier transmission. **e** and **f** Δ = 9Ω and **M**_**I**_ set with three equal-amplitude components and positive/negative phases on the carrier transmission. Complex amplitudes of the entries of the matrices are denoted by the color map. **b**, **d**, **f**, **h** Measured output amplitudes on the synthetic lattice from the settings in (**a**), (**c**), (**e**) and (**g**), respectively. *c*_*j*_ represents the *j*-th column of **G**, and *x*_*k*_ denotes the input at the *k*-th lattice site. Error bars denote the data range from five measurements. Insets in (**b**), (**d**), (**f**) and (**h**) are the corresponding results from the theoretical calculations.

For the first MVM experiment, we set the laser frequency at the center optical resonance (Δ = 0), and remove the carrier transmission, thus providing an input vector ${{{{{\bf{x}}}}}}={({{{{\mathrm{..}}}}}.,0,{x}_{-1},0,{x}_{1},0,{{{{\mathrm{..}}}}}.)}^{{{{{{\rm{T}}}}}}}$ where *x*_−1_ = -*x*_1_ (Fig. 3a). These two input components couple to the 25 sites on the synthetic lattice through the non-local frequency conversions, which add up to a symmetric amplitude distribution at the output (Fig. 3b). The difference between the experimental results and the theory is mostly due to the background transmission of the optical cavity (not included in the theoretical model), which becomes pronounced at high cavity modulation depth as the input optical field is scattered to many sidebands. Next, we tune the DC bias to generate an input ${{{{{\bf{x}}}}}}={({{{{\mathrm{..}}}}}.,0,{x}_{-1},{x}_{0},{x}_{1},0,{{{{\mathrm{..}}}}}.)}^{{{{{{\rm{T}}}}}}}$, where |*x*_0_| = |*x*_1_|and arg(*x*_0_) = −arcsin(*x*_1_/*a*_in_) (Fig. 3c). Such additional component *x*_0_ is also coherently scattered to the entire frequency lattice and induces the interference with the pattern formed by *x*_±1_, leading to an asymmetry in Fig. 3d. To demonstrate the coherence of the full range “edge-to-edge” connections, we then align the laser frequency to the 9th site (Δ = 9Ω), and set **M**_**I**_ to produce an input ${{{{{\bf{x}}}}}}={({{{{\mathrm{..}}}}}.,0,{x}_{8},{x}_{9},{x}_{10},0,{{{{\mathrm{..}}}}}.)}^{{{{{{\rm{T}}}}}}}$ with |*x*_8_| = |*x*_9_| = |*x*_10_|. The DC bias on **M**_**I**_ is switched between the phase relation of either arg(*x*_9_) = arcsin(*x*_10_/*a*_in_) (Fig. 3e) or arg(*x*_9_) = −arcsin(*x*_10_/*a*_in_) (Fig. 3g). From the measured output amplitude patterns, we observe the suppression (Fig. 3f) and revival (Fig. 3h) of the negative-order sites near the opposite edge by only phase-flipping of the carrier transmission, a strong indicator of the built-in long-range phase coherence with the frequency-domain MVM operations.

### Concatenated phase modulator networks

**Fig. 4:**
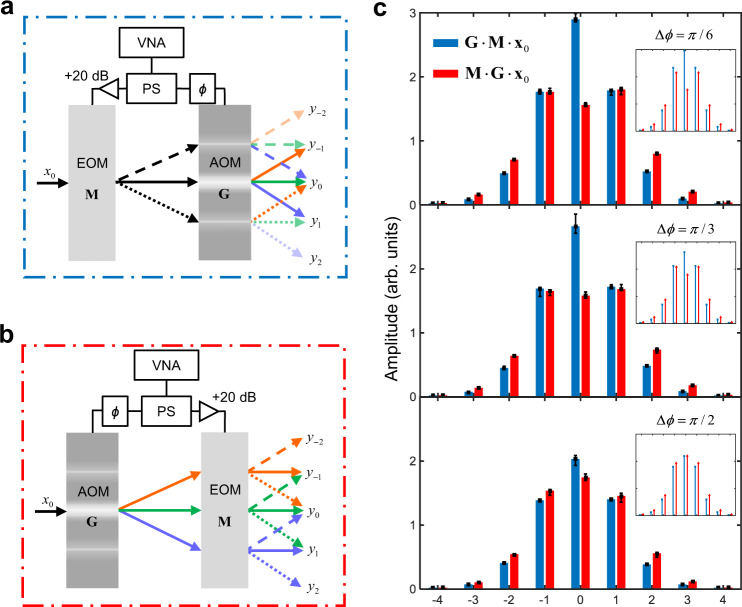
Concatenations of phase modulators. **a** Setup of concatenated broadband electro-optic phase modulator (EOM, **M**) and our device (AOM, **G**) driven by the same vector network analyzer (VNA). A laser input at Δ = 0 (**x**_0_) goes through the EOM first and then the AOM, **G**∙**M**∙**x**_0_. **b** Setup of the concatenation in the reverse order, **M**∙**G**∙**x**_0_. Orange, green and blue lines represent the −1st, 0th (carrier) and 1st harmonic signals generated by the AOM. Solid, dotted and dashed lines represent the carrier and ±1st sideband signals by the EOM. *y*_*k*_ denotes the output of the *k*-th synthetic lattice sites. PS: RF power splitter; +20 dB: RF amplifier with 20 dB gain; *ϕ*: RF phase shifter. **c** Contrast of the output between the two concatenation orders **G**∙**M**∙**x**_0_ (blue) and **M**∙**G**∙**x**_0_ (red) with varying phase Δ*ϕ* at the two modulations. Error bars are plotted from five measurements. Insets are the corresponding results from the theoretical calculations.

Practical optical computations using integrated photonics require programmability with a high degree of freedom to allow on-chip optimization processes such as backpropagation training in a neural network. For the frequency-domain computing architecture, a straightforward approach to increase independently tunable parameters is to concatenate multiple modulators controlled by separate electronics. To this end, we notice the general phase modulations in the full parametric space, including the broadband elements, constitute a non-abelian (noncommutative) group $< G,\cdot >$, where the concatenation of modulators defines the binary operation “ ∙” with the matrix-matrix multiplication. The noncommutativity can manifest as nonreciprocal frequency conversions resulted from the coupling phase anisotropy and the non-unitarity (see Supplementary Note 11). To demonstrate the feasibility of the concatenation architecture, we implement two elements of the group ${{{{{\bf{G}}}}}},{{{{{\bf{M}}}}}}\in < G,\cdot >$ with our device and a broadband EOM, driven by the same microwave tone. We cascade them in both **G** ∙ **M** (Fig. 4a) and **M** ∙ **G** (Fig. 4b) orders and probe the responses with a laser frequency Δ = 0. Since broadband EOMs typically have a low modulation efficiency with *V*_π_ at a few volts, we choose the RF drive at 2.903 GHz and add a 20 dB amplifier to the driving arm of the EOM to reach comparable modulation indices. In this scheme, the RF driving phases at both **G** and **M** count as independently programmable parameters in addition to the modulation depth. To reflect this increased programmability, we use a tunable RF phase shifter which controls the temporal delay between the two modulations. Figure 4c compares the measured output amplitudes for both the concatenation orders with varying modulation phase delay. The error bars at the 0th synthetic lattice site are slightly longer than the other sites due to a weak time-varying beating signal in the local oscillator (LO) in our heterodyne measurement (see Methods). Nevertheless, the contrast of the results clearly shows the driving phase control of the noncommutative frequency conversions, featuring the non-abelian algebraic structure.

## Discussion

In conclusion, we have demonstrated an ultra-efficient nanophotonic acousto-optic modulator on the hybrid AlN-on-SOI platform that performs large-scale, complex-valued MVM in a fully connected fashion. We reveal the advantages of high scalability and long-range phase coherence associated with the MVM in the synthetic frequency dimension. In applications of optical computing, our device exploits the spectral degree of freedom to enable quadratic and cubic scaling of operations with the length of the operands in the linear layer of matrix-vector and matrix-matrix multiplications, respectively^25^, while occupying a compact footprint. This is in stark contrast to conventional wavelength division multiplexing where only the data bandwidth increases with independent channels. Further considerations on the engineering of the integrated electromechanical transducer will allow the generation of multiple harmonic tones at *n*Ω through Fourier synthesis of an arbitrary periodic modulation waveform^38,39^ to facilitate MVM operations with enhanced programmability (see Supplementary Note 12). While our demonstrations have not experimentally realized a practical optical computing system, our work opens the door to a disruptively new silicon-based optical computing architecture that is scalable with significantly increased operand vector length in compact footprints, as envisioned in Supplementary Note 13. Such frequency-domain scheme also extends naturally to scalable quantum computing by incorporating optical quantum sources such as single-photon parametric down-conversion, spontaneous four-wave mixing^40,41^, and microwave qubit transduction^35^, where the efficient dynamic modulators facilitate frequency-bin logic gates for high-dimensional photonic qudits^42^.

## Methods

### Device fabrication

The substrate was prepared by sputtering 320-nm thick polycrystalline AlN on silicon-on-insulator (SOI) wafer with 220 nm Si layer (grown by OEM group). Before patterning the structure, a layer of silicon dioxide (SiO_2_) was deposited as a hard mask by plasma-enhanced chemical vapor deposition. We first patterned the window for silicon photonic structures by electron-beam lithography (EBL) with positive ZEP520A resist (developer: amyl acetate), which was subsequently transferred to the SiO_2_ hard mask by fluorine-based inductively-coupled-plasma etching (ICP-F). The exposed AlN in the window was removed by another step of chlorine-based inductively-coupled-plasma etching (ICP-C) with Cl_2_/BCl_3_/Ar chemistry followed by nitrogen plasma cleaning. The etching time was precisely controlled to remove only the targeted AlN layer. The silicon photonic structures, including the one-dimensional photonic crystal cavity, grating couplers and waveguide, were patterned using aligned EBL and negative hydrogen silsesquioxane (HSQ) resist (developer: tetramethylammonium hydroxide, TMAH), followed by an ICP-C etching with Cl_2_ plasma. Another round of aligned EBL (resist: ZEP520A) and ICP-C was applied to etch through the free-edge reflector and the releasing windows for the final releasing step. The remaining positive resist and on-top oxide (including the remaining HSQ) were removed by N-Methyl-2-pyrrolidone (NMP) and buffered oxide etchant, respectively. We then patterned the IDT using a third-time aligned EBL (resist: ZEP520A), followed by electron-beam evaporation of aluminum and lift-off in NMP. Finally, the device was released by vapor hydrofluoric acid, which removed the buried oxide layer to achieve the suspended structure.

### Experimental characterizations

We used homodyne and heterodyne measurements to characterize the modulation in terms of the microwave-to-optical transduction signal and the harmonic signal generations, respectively (see Supplementary Note 5). The optical input with tunable frequency was provided by coupling a continuous-wave laser (TSL-710; Santec) to the on-chip input grating coupler. The IDT was driven by the transmitter port (Port 1) of a calibrated vector network analyzer (E8362B; Agilent) with tunable RF frequency and power. The optical output of the device was collected from the output grating coupler, which was subsequently sent to a 2 × 2 optical switch (OSW22-633E; Thorlabs) with one port connected to a low-speed photodetector (2053-FC; Newport) for the direct-current (DC) transmission measurements, and the other to a serial connections of erbium-doped fiber amplifier (PM-LNHPFA-15; PriTel), tunable optical bandpass filter (OTF-910; Santec) and high-speed photodetector (1544-B; Newport) for the characterizations of the frequency conversions. In the homodyne measurements, the down-converted signal from the high-speed photodetector was sent to the receiver port (Port 2) of the vector network analyzer. The *S*_21_ trace then measured the spectrum of the microwave-to-optical transduction signal corresponding to the generated first-order beating note in the detected optical output. We used the spectrum of *S*_21_ to identify the mechanical resonance frequencies with enhanced modulation efficiencies (see Supplementary Note 6). For the heterodyne measurements, the laser beam was split by a 10/90 benchtop optic coupler, in which the 90% part was used as a LO. The LO was sent through an acousto-optic frequency shifter (TEM-110-10-55-2FP; Brimrose) and up-shifted by 102.9 MHz. The LO was combined with the optical output from our device at a 50/50 benchtop optic coupler, generating a set of spectrally resolved beating notes proportional to the modulation-induced harmonic signals, respectively. We measured the amplitudes of these harmonic signals by a real-time spectrum analyzer (RSA5126B; Tektronix), and read out the results of the MVM accordingly (see Supplementary Note 5).

### Reporting summary

Further information on research design is available in the Nature Research Reporting Summary linked to this article.

## Supplementary information

Supplementary Information

Reporting Summary

## Data Availability

All the data that support the findings of this study are available within the paper and the supplementary information.
