# Supplementary material for: Enabling scalable optical computing in synthetic frequency dimension using integrated cavity acousto-optics
Source: Nat Commun. 2022 Sep 15;13:5426. doi: 10.1038/s41467-022-33132-z (PMC9477821; doi:10.1038/s41467-022-33132-z)
Supplement: Supplementary file 1 — Supplementary Information [file 41467_2022_33132_MOESM1_ESM.pdf]

Supplementary Materials for

Scaling optical computing in synthetic frequency dimension using integrated cavity acousto-optics

Han Zhao[1*]†, Bingzhao Li[1]†, Huan Li[1], and Mo Li[1,2*]

[1]*Department of Electrical and Computer Engineering, University of Washington, Seattle, WA 98195, USA*

[2]*Department of Physics, University of Washington, Seattle, WA 98195, USA*

†These authors contributed equally to the work

*Email: hzhao89@uw.edu; moli96@uw.edu

Supplementary Note 1. Device parameter and fabrication flow

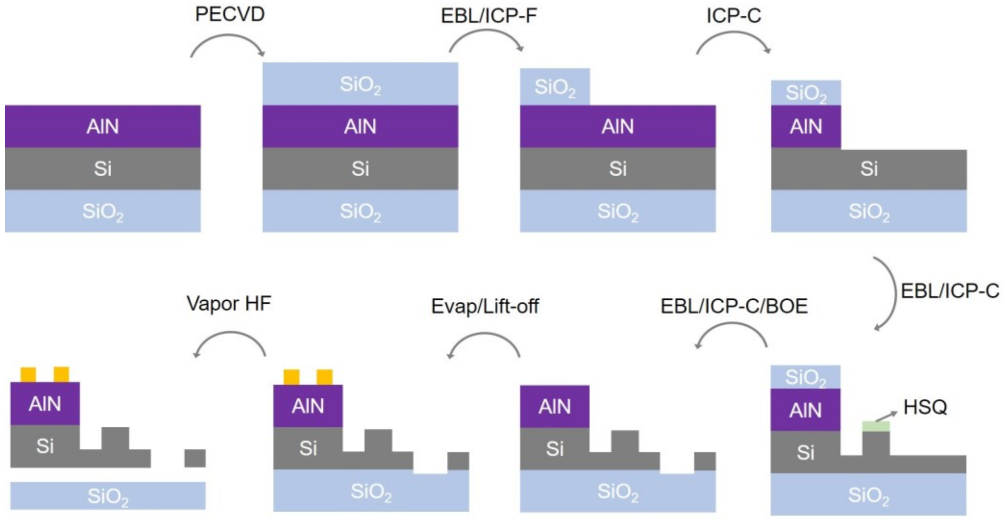

Fig. S1: Fabrication flow for the cavity acousto-optic modulator on hybrid AlN-on-SOI platform. PECVD: plasma-enhanced chemical vapor deposition; EBL: electron-beam lithography; ICP-F: fluorine-based inductively-coupled-plasma etching; ICP-C: chlorine-based inductively-coupled-plasma etching; HSQ: hydrogen silsesquioxane; BOE: buffered oxide etchant; Evap: electron-beam evaporation; Vapor HF: vapor hydrofluoric acid.

Table S1 Parameters of the fabricated cavity acousto-optic modulator

Thickness of AlN (nm)	320	Thickness of Si (nm)	220
Thickness of buried oxide (μm)	3	Width of waveguide (nm)	550
Strip height of waveguide (nm)	150	Height of Si sleeve (nm)	70
Periodicity of photonic crystal (nm)	350	Depth of holes (nm)	150
Length of rectangular holes (nm)	375	Maximum hole width (nm)	180
Minimum hole width (nm)	80	Number of tapering	25
Thicknesses of Al electrodes (nm)	220	Periodicity of IDT (μm)	3
Width of IDT finger (nm)	375	Total IDT length (μm)	150
Distance from IDT to waveguide (μm)	10	Distance from waveguide to free-edge reflector (μm)	2.4

Supplementary Note 2. Analysis of the piezoelectrically transduced mechanical modes

The IDT patterned on the heterogeneous AlN/Si region is used to resonantly excite multiple mechanical modes, which have very distinct acousto-optic modulation efficiencies on the nanophotonic cavity. To understand the relation between the acousto-optic modulations and these mechanical modes, we performed the numerical simulations (COMSOL Multiphysics 5.5) and show in Fig. S2 the displacement fields in the suspended AlN/Si layer, corresponding to the resonances measured by the IDT S_{11} response. The mechanical modes that are of the interest in the main text are the fundamental Lamb mode (large out-of-plane displacement) at ~800 MHz and the fundamental compressional mode (large in-plane displacement) at ~2.9 GHz. Because of the long wavelengths, these two modes strongly couple to the 70-nm Si membrane and the optical nanobeam cavity

therefore can induce strong phase modulations. We characterize the acousto-optic modulations by measuring the microwave-to-optical transduction signal S_{OE} and exploit these modes for the frequency-domain matrix-vector multiplications.

Our IDT with the split-finger design can also excite higher-order mechanical modes with odd-number modal orders. In the 500 MHz to 8 GHz spectrum, Mode III is the 3rd-order Lamb mode; Mode V is the 5th-order compressional mode; Mode IV is the 3rd-oder Love mode; and Mode VII is the 3rd-oder AlN/Si Lamb-compression hybrid mode. These higher-order modes are associated with significantly reduced wavelengths which increase the mechanical power dissipation and decrease the modal overlap between the mechanical modes and the optical cavity field (so weaker optomechanical coupling). Consequently, the higher-order mechanical modes have negligible acousto-optic phase modulation efficiencies (compared to the fundamental orders) and dominantly contribute to thermo-optic tuning of the optical cavity resonance. In addition, we also observe the excitations of a symmetric breathing mode (VI). Although this breathing mode shows a larger piezoelectric transduction efficiency, it does not couple to the acoustic wave in the silicon membrane (in Lamb mode) due to the mismatch of the modal symmetry, therefore has no phase modulation effect.

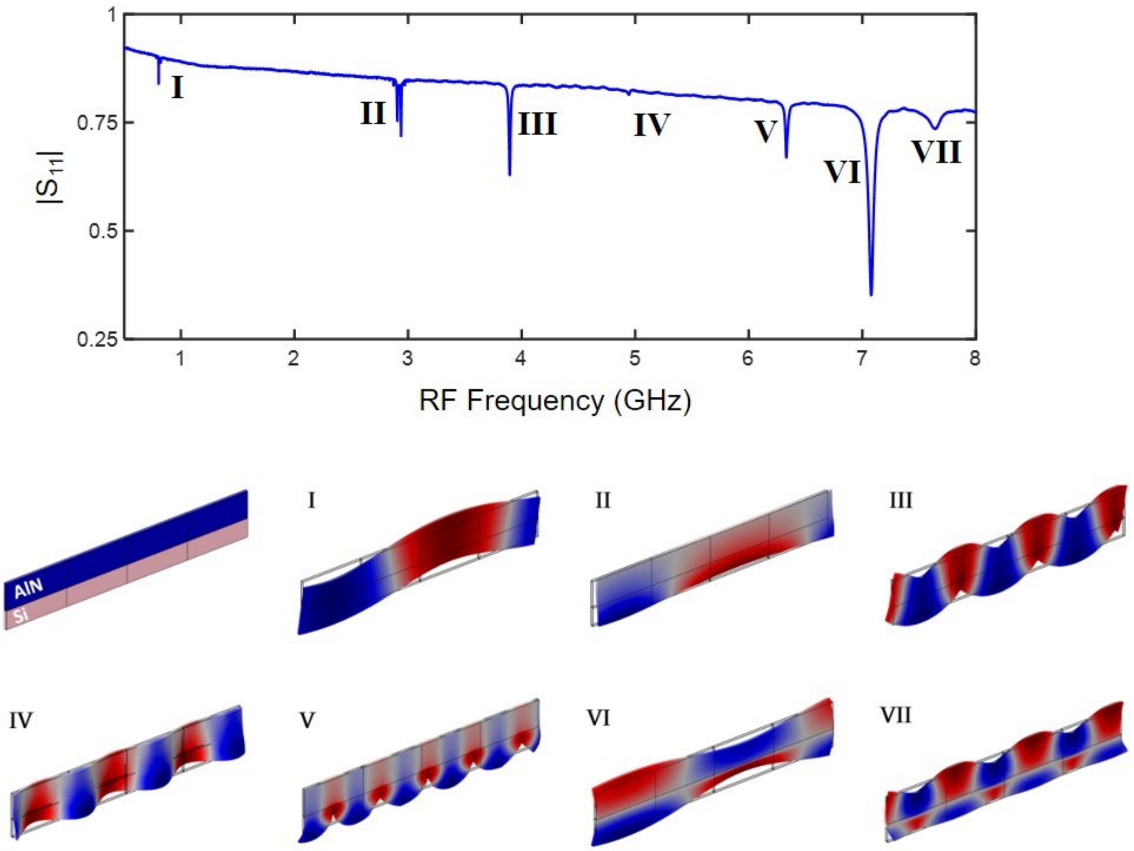

Fig. S2: Spectrum of the mechanical modes in the AlN/Si region when the IDT is driven in the RF frequency range of 500 MHz – 8 GHz. I-VII show the simulated distributions of the displacement fields in a single IDT period (3 μm), which correspond to the resonance modes in the experimentally measured IDT spectrum, respectively.

Supplementary Note 3. Analytical solutions to the intra-cavity photon dynamics

The intra-cavity photon dynamics under the acousto-optic modulation (equation (1) in the main text) has explicit solutions when a single microwave tone is applied on the IDT, i.e. $\hat{f}(t) = \cos(\Omega t + \phi)$. The solution provides the insight of the physics under our interrogation. Therefore, before introducing our experimental characterizations, we show here the derivation of the analytical solutions of both the intra-cavity and the output optical fields.

We rewrite the dynamics with $\hat{f}(t) = \cos(\Omega t + \phi)$ as

$$\dot{a}(t) = i\Delta a(t) - i\beta \cdot \Omega \cdot \cos(\Omega t + \phi)a(t) - \kappa a(t)/2 + \sqrt{\kappa_{\text{ex}}}a_{\text{in}}(t), \quad (1)$$

where $a(t)$ and $a_{\text{in}}(t)$ are the intra-cavity and input optical fields, respectively; κ and κ_{ex} denote the total optical cavity decay rate and external coupling rate, respectively; $\Delta = \omega_{\text{p}} - \omega_0$ is the detuning of the input laser angular frequency ω_{p} from the cavity center frequency ω_0; Ω, ϕ are the frequency and phase of microwave drive applied to the IDT, respectively; $\beta = 2g_{\text{om}}/\Omega$ is the modulation index; and g_{om} is the total optomechanical coupling. By the transformation $a(t) = \alpha(t)\exp[-i\beta\sin(\Omega t + \phi)]$, we obtain

$$\dot{\alpha} = (i\Delta - \kappa/2)\alpha + \sqrt{\kappa_{\text{ex}}}e^{i\beta\sin(\Omega t+\phi)}a_{\text{in}}. \quad (2)$$

Using Jacobi–Anger expansion

$$\exp[i\beta\sin(\Omega t + \phi)] = \sum_k J_k(\beta)\exp[ik(\Omega t + \phi)], \quad (3)$$

where $J_\nu(x)$ is the Bessel function of the first kind, we decompose equation (2) in Fourier series $\alpha(t) = \sum_k \alpha_k \exp(ik\Omega t)$, and obtain

$$ik\Omega\alpha_k = (i\Delta - \kappa/2)\alpha_k + J_k(\beta)e^{ik\phi}a_{\text{in}}, \quad (4)$$

which leads to

$$\alpha_k = e^{ik\phi}J_k(\beta)\frac{a_{\text{in}}\sqrt{\kappa_{\text{ex}}}}{i(-\Delta + k\Omega) + \kappa/2}. \quad (5)$$

The dynamics of the intra-cavity optical field can then be expressed as

$$\begin{aligned} a(t) &= e^{-i\beta\sin(\Omega t+\phi)} \cdot \sum_k \alpha_k \exp(ik\Omega t) \\ &= \sum_n e^{-in\Omega t}\sum_k J_{n+k}(\beta)J_k(\beta)e^{-in\phi}\frac{\sqrt{\kappa_{\text{ex}}}a_{\text{in}}}{i(-\Delta + k\Omega) + \kappa/2}. \end{aligned} \quad (6)$$

Thereby, the optical output at the exit facet of the end-coupled optical nanobeam cavity can be explicitly calculated as

$$a_{\text{out}}(t) = \sum_n e^{-i(\omega_{\text{p}}+n\Omega)t}\sum_k J_{n+k}(\beta)J_k(\beta)e^{-in\phi}\frac{\kappa_{\text{ex}}a_{\text{in}}}{i(-\Delta + k\Omega) + \kappa/2}. \quad (7)$$

At high modulation index $\beta >> 0$, the Bessel functions $J_\nu(\beta)$ are non-vanishing for $\nu >> 1$, leading to large amplitudes at higher order harmonic signals. Hence, the output optical signal for a single-frequency input at ω_{p} can be thought of as a compositional baseband of many RF harmonics modulated by the optical carrier frequency ω_{p}. In the following, we show our method to experimentally characterize each of the RF harmonics in the optical output.

Supplementary Note 4. Boundedness of the effective vector space of the matrix-vector multiplications in the synthetic frequency dimension

It is important to have bounded vector space when performing matrix-vector multiplications in the synthetic frequency, such that severe optical energy loss to unwanted sidebands can be prevented. For cascaded modulator systems that involve *only* broadband modulators and/or resonating modulators with spectrally uniform modes (such as ring resonators), the vector space will spread out infinitely along the cascaded chain. However, our approach uses the acousto-optic modulation of a single nanophotonic cavity mode where the coherent frequency conversions only happen among a finite number of sidebands bounded near the cavity resonance frequency. The size of the non-trivial vector space, i.e., the number of relevant sidebands that participate in the frequency conversions, can be determined by the boundary of nonzero entries of the optomechanical coupling matrix **G** (equation (3) in the main text), since the zero terms outside the boundary denote the vanishing of the indexed sideband couplings. As shown in Fig. S3, this boundary is restricted by the modulation index β, which can be controlled by the driving power.

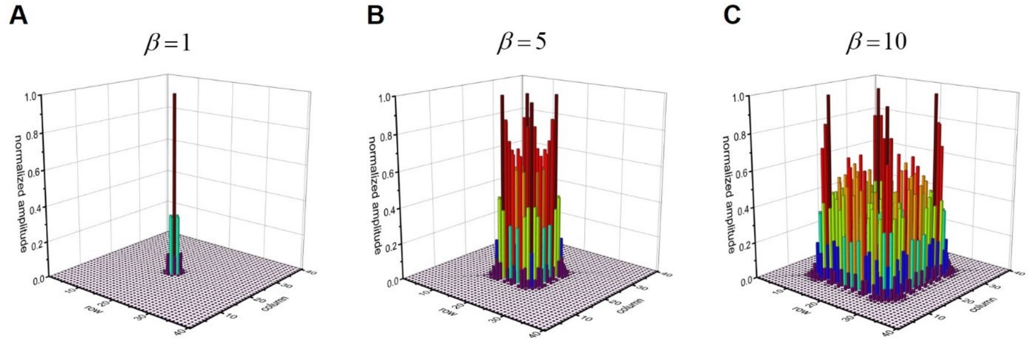

Fig. S3: Boundary of the optomechanical coupling matrix at various modulation indices β. (**A**) β = 1. (**B**) β = 3. (**C**) β = 10. The matrices are self-normalized by their maximum entries, respectively. The length of the effective vector space is constrained within the boundary of the optomechanical coupling matrix.

To understand quantitatively, we notice the coupling coefficient from the n-th sideband to the m-th sideband in the is

$$g_{mn} = \sum_k J_{m-n+k}(\beta) J_k(\beta) e^{-i(m-n)\phi} \frac{\kappa_{\text{ex}}}{i(k-n)\Omega + \kappa/2}, \quad (8)$$

where $J_v(\beta)$ is the v-th order Bessel function of the first kind. Because of the sideband-resolved resonance feature ($\Omega > \kappa$), the above summation can be well approximated by a few terms around $k = n$ while the other terms vanish. Meanwhile, with finite β, the value of $J_v(\beta)$ is only non-trivial for the first several orders, that is, the terms in the summation that contribute to the frequency conversion have upper bounds for both k (n) and $m - n + k$. Hence, the frequency conversions only happen for optical frequencies at a finite number of sidebands (n) near the cavity resonance, and the range of the frequency coupling ($m - n$) is restricted by the modulation index. These two factors lead to the truncation of the effective vector length in our MVM operations. For the same reasons, in a cascaded network of single-resonance modulators, the size of the vector space is also naturally bounded, and is determined by the frequency span (modulation index) at the last modulator of the cascaded chain. Therefore, by controlling the driving power applied to

the modulators such that the modulation indices increase along the cascading order, we are able to regulate the size of the effective vector space for performing MVM operations with a finite basis, while minimizing the optical energy loss to irrelevant sidebands.

Supplementary Note 5. Experimental characterization of the cavity acousto-optic modulator

We used homodyne and heterodyne measurement schemes to characterize the modulation in terms of the microwave-to-optical transduction signal (S_{OE}) and the harmonic signal generations (Figs. 2-4 in the main text), respectively. Fig. S4 shows the experimental setup. The interdigital transducer (IDT) was driven by the transmitter port (Port 1) of a calibrated vector network analyzer (VNA) with tunable RF frequency and power output. The optical input was realized by coupling a continuous-wave (CW) laser to the on-chip grating coupler through a polarization-maintained fiber, and the output was collected from the output grating coupler by another aligned fiber. We measure the spectra of the direct-current (DC) transmitted optical power (Fig. 1c, Fig. 2b and Fig. 2c) by switching the optical output from our device to a low-speed photodetector (LPD) while sweeping the laser frequency. The high-frequency components of the transmitted optical signal are interrogated by a square-rule high-speed photodetector (HPD) with a bandwidth of 12 GHz, which down-converts the beating notes of the detected optical signal to corresponding RF voltages.

In the homodyne branch, we switched off the acousto-optic frequency shifter (AOFS) and sent the down-converted signal from the HPD to the receiver port (Port 2) of the VNA. The S_{21} parameter of VNA then measured the HPD-generated RF signal at the driving microwave frequency normalized by the input RF complex amplitude, which is proportional to the first-order optical beating note in $a_{\text{out}} \cdot a_{\text{out}}^*$, where a_{out} is the output field in equation (7), i.e.,

$$S_{21} \propto \sum_n a_n \cdot a_{n-1}^* . \tag{9}$$

We note it is only possible for this S_{21} to take nonzero value if the electromechanically transduced acoustic wave modulates the optical field. It is therefore also named the microwave-to-optical transduction signal ($S_{OE} = S_{21}$). In most of previous works where the modulation index is small (only a_0, a_1 and a_{-1} are relevant), S_{OE} can be simplified to $S_{\text{OE}} \propto a_0 a_{-1}^* + a_1 a_0^*$, which is widely used as the metric to the modulation depth and bandwidth when the laser frequency is tuned at the red sideband ($\Delta = -\Omega$, $S_{\text{OE}} \propto a_1$) or the blue sideband ($\Delta = \Omega$, $S_{\text{OE}} \propto a_{-1}^*$) for sideband-resolved acousto-optic systems. Another functionality of our homodyne measurements is to identify the center optical resonance frequency ω_0 because S_{OE} equals zero at exactly zero detuning $\Delta = 0$ and has a large gradient in the vicinity. We used the traces of the $|S_{21}|$ center local minimum shown in Fig. S5 to characterize the thermo-optic shift induced by the acoustic wave. For our acousto-optic modulator with high modulation index, however, S_{OE} is a complicated composition contributed from the frequency conversions between the adjacent sidebands, and therefore cannot fully characterize the dynamic phase modulation. This necessitates the heterodyne measurements that can spectrally resolve all the harmonic signals received by the HPD.

In the heterodyne branch, we drive the AOFS at an angular frequency $\omega_\mu = (2\pi) \cdot 103$ MHz, which shifts the optical frequency of the local oscillator (LO) to $\omega_\mu + \omega_p$. When

combined with the optical output from our acousto-optic modulator, the signal received at the HPD can be written as (by ignoring the high frequency components)

$$U_{\text{hetero}} \propto \left(c_0 e^{-i(\omega_\text{p}+\omega_\mu)t} + \sum_n a_n e^{-i(\omega_\text{p}+n\Omega)t}\right)\left(c_0 e^{-i(\omega_\text{p}+\omega_\mu)t} + \sum_n a_n e^{-i(\omega_\text{p}+n\Omega)t}\right)^* . \quad (10)$$

The down-converted RF voltage contains the frequency components at ω_μ - $n\Omega$ which have the amplitudes proportional to the corresponding n-th harmonic signals in the optical output by a factor of the LO amplitude h_0, i.e.

$$| u_{\omega_\mu - n\Omega} | \propto h_0 \, | a_n | . \quad (11)$$

Therefore, by mapping out all the RF frequency components in a real-time spectrum analyzer (RSA), we can capture all the amplitudes of the harmonic generations induced by the acousto-optic modulation. The heterodyne measurements were used to obtain the experimental results in Figs. 2d, e, Fig. 3 and Fig. 4.

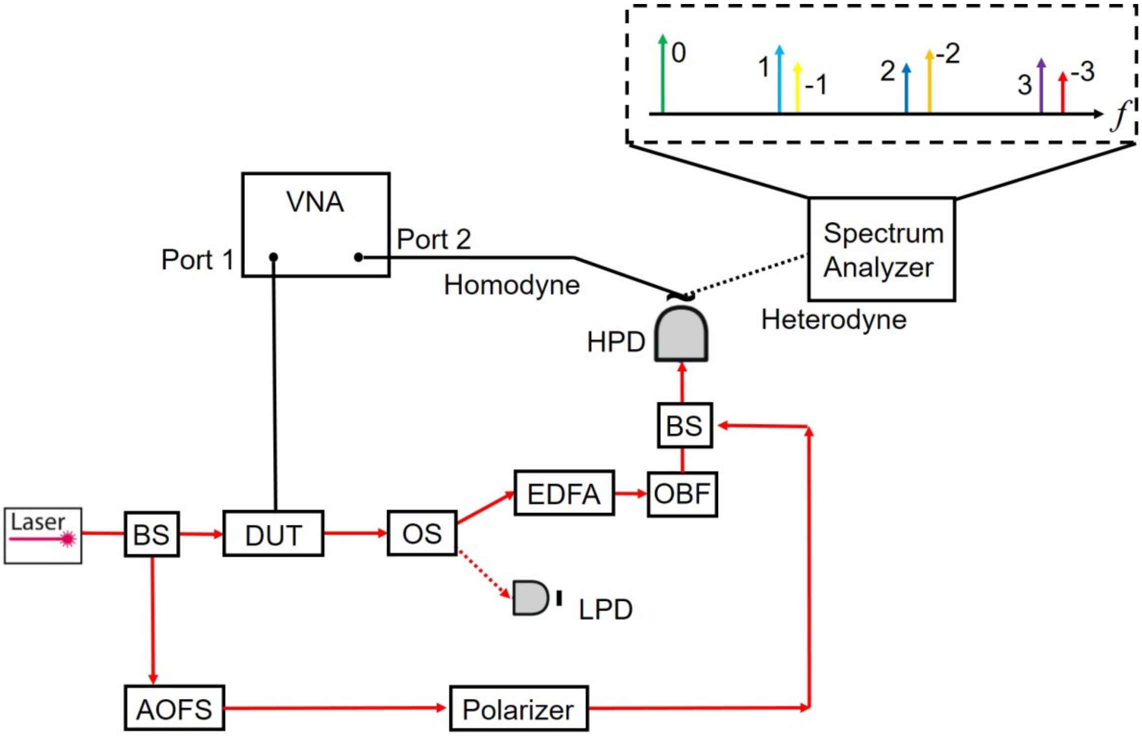

Fig. S4: Experimental setup for the homodyne/heterodyne measurements. BS: optical in-line beam splitter; DUT: device under test; AOFS: fiber acousto-optic frequency shifter; OS: optical switch; LPD: low-speed photodetector; EDFA: erbium-doped fiber amplifier; OBF: tunable optical bandpass filter; HPD: high-speed photodetector (12-GHz bandwidth); VNA: vector network analyzer.

Supplementary Note 6. RF spectra of the microwave-to-optical transduction

We use the homodyne measurements to characterize the spectrum of the acousto-optic modulation and the associated thermo-optic shift for the RF drive at varying RF tone. Fig. S5 shows the spectrum of the measured microwave-to-optical transduction signal at the mechanical resonances, including the prominent fundamental Lamb mode and compressional mode. The acoustic resonator formed by the free-edge reflector gives rise to a series of resonances in the IDT bandwidth. By mapping out the RF spectrum of S_{OE},

we were able to identify the on-resonance microwave tones that can induce the most efficient modulation for each mechanical mode. The laser frequency is swept around the intrinsic nanophotonic cavity resonance to probe S_{OE} at all sidebands.

For the fundamental Lamb mode excitation at ~800 MHz, we observed the appearance of S_{OE} at multiple resolved sidebands even at a low RF power of -16dBm. In particular, the 803 MHz drive with an RF bandwidth of 1.3 MHz induces a significantly increased number of sidebands, consistent with the most pronounced electromechanical conversion efficiency measured from S_{11}. This RF tone thus facilitates the resonantly enhanced acousto-optic modulation, by which we demonstrated the scalable MVM at a large-scale synthetic frequency lattice. The minimum at zero laser frequency detuning indicates a constant optical center resonance frequency (no pronounced thermo-optic shift) at the -16 dBm RF power (Fig. S5A). For the fundamental compressional mode excitation at ~2.9 GHz, the highest modulation efficiency is achieved at 2.903 GHz, where the optical mode overlaps with the anti-node of the acoustic resonator. The 2.935 GHz resonance has a weaker modulation because the optical mode primarily overlaps with the node of the mechanical standing wave. The acoustic resonances in this frequency range are subject to more mechanical power dissipation, evidenced by the increased linewidth of 7.5 MHz. As a result, a substantial red shift of the optical center resonance frequency can be observed at the RF power of -6 dBm, shown by the trace of the local minimum in the middle of Fig. S5B. As mentioned in Supplementary Note 2, other higher-order mechanical modes have much reduced modulation efficiency. As an example, we show the spectrum of S_{OE} for the 5th-order compressional mode in Fig. S5D, which is barely measurable even at the RF power of 0 dBm. We remark that the excitations of higher-order mechanical modes, while contributing negligibly to the dynamic phase modulation, can function as thermo-optic resonance tuning, beneficial for aligning the operation frequencies in concatenated modulator networks.

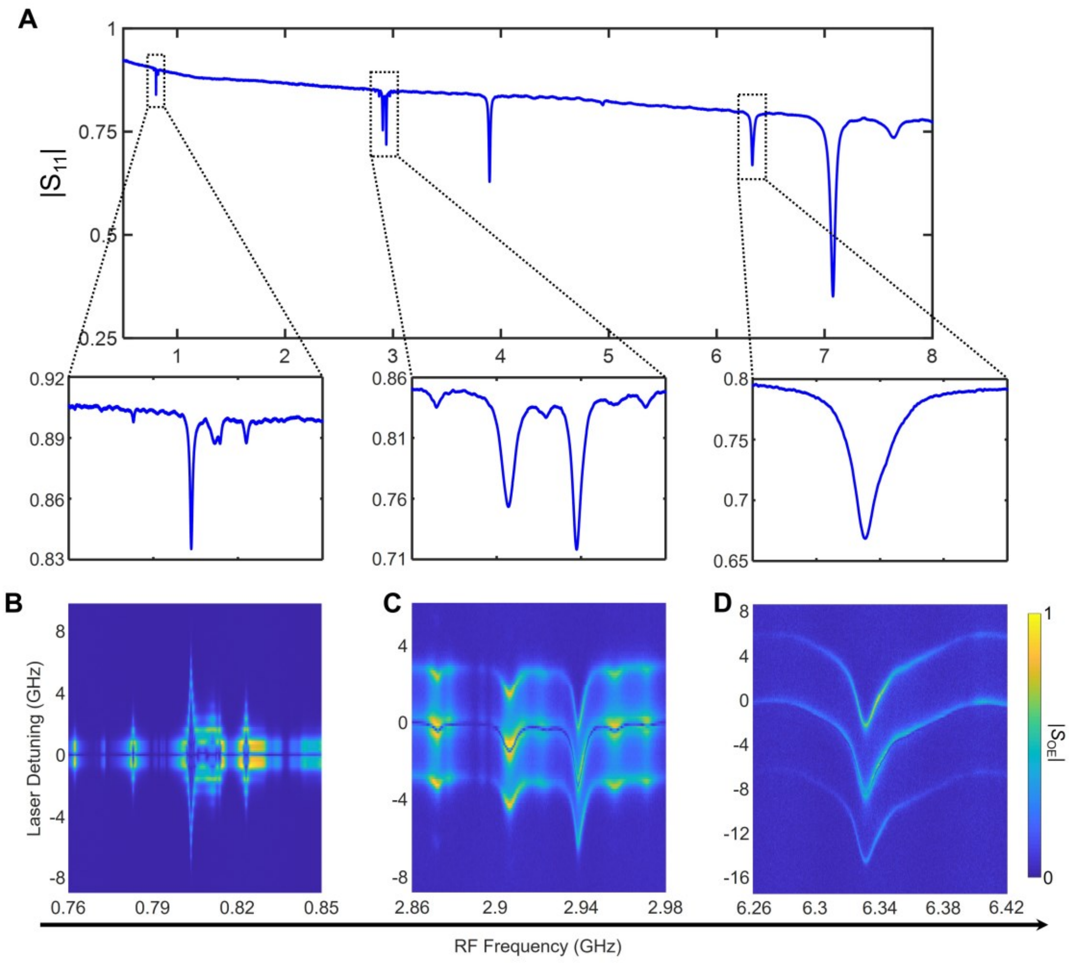

Fig. S5: Characterizations of the microwave-to-optical transduction. (**A**) RF spectrum of the IDT reflection coefficient $|S_{11}|$. The zoom-in reflection spectra correspond to the excitations of the fundamental Lamb mode, fundamental compressional mode and 5th-order compressional mode, respectively. (**B**) Amplitude of S_{OE} as a function of the RF driving frequency and the laser detuning for the fundamental Lamb mode. The RF power is fixed at -16 dBm. (**C**) Amplitude of S_{OE} for the fundamental compressional mode at the RF power of -6 dBm. (**D**) Amplitude of S_{OE} for the 5th-order compressional mode at the RF power of 0 dBm.

Supplementary Note 7. Determining the modulation index from the spectra of optical transmittance

One of the consequences of the high acousto-optic modulation index is the generation of multiple sidebands in the optical transmission spectrum. In our sideband resolved system, we can extract the modulation index by fitting the measured spectral features of the split sidebands. To see this, here we show the exemplary fitting results under single microwave tone drives at 2.903 GHz and 803 MHz.

The theoretical values of the DC transmittance can be derived from equation (7) and takes the form

$$\left\langle a_{\mathrm{out}} \cdot a_{\mathrm{out}}^{*} \right\rangle = \sum_{n} \left| J_{n}(\beta) \frac{\kappa_{\mathrm{ex}} a_{\mathrm{in}}}{i(-\Delta + n\Omega) + \kappa/2} \right|^{2} . \tag{12}$$

We use equation (12) to fit the measured spectra of the optical transmittance at varying RF driving power (Figs. 2b,c in the main text), where the parameters κ, κ_{ex}, Ω, a_{in} were fixed and only the modulation index β is varied to reproduce the spectral features. Fig. S6 shows the fit of a transmittance spectrum at Ω = 2.903 GHz and 2 dBm RF power. From the agreement between the measured and calculated spectra, we deduced a modulation index $\beta = 2.15$.

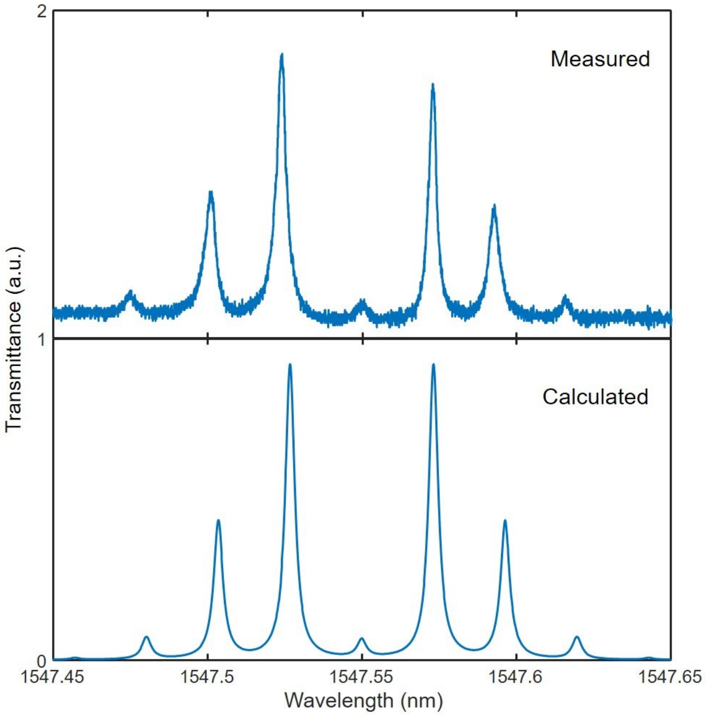

Fig. S6: Measured optical transmittance spectrum and the corresponding fit curve with β = 2.15. The microwave drive is at Ω = 2.903 GHz and 2 dBm RF power (upper). The fit curve is calculated by equation (12) (lower). The measured spectrum is shifted up by 1 unit.

For large modulation depth observed at Ω = 803 MHz, the transmission eigenstate distributes to all of the sidebands spanning a wide spectral range and is superposed by the non-uniform background transmission. Nonetheless, we expect a good characterization of the spectral features by a proper fit parameter β. Fig. S7 shows the fit of the measured transmittance spectrum at Ω = 803 MHz and -7 dBm RF power. This corresponds to the maximum modulation index of β = 22.9 obtained before the onset of electromechanical nonlinearity, where the modulation index ceases to increase proportional to the square-root of the RF power.

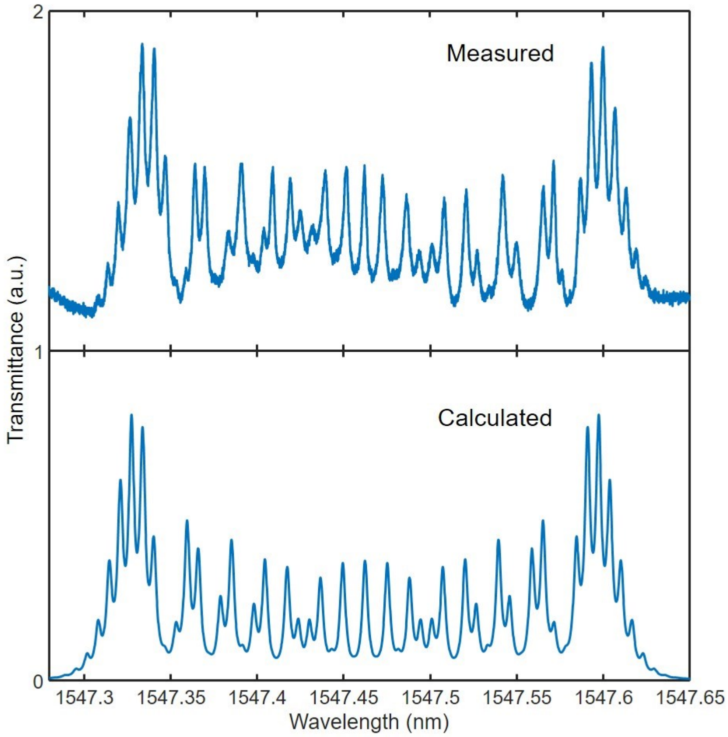

Fig. S7: Measured optical transmittance spectrum and the corresponding fit curve with β = 22.9. The microwave drive is at Ω = 803 MHz and -7 dBm RF power (upper). The fit curve is calculated by equation (12) (lower). The measured spectrum is up shifted by 1 unit.

Supplementary Note 8. Optical spectra of the high-order harmonic signal generations

We used the heterodyne measurement setup to characterize all the harmonic signal generations whose amplitudes are proportional to the corresponding frequency components in the converted RF voltage at the HPD. The theoretical results of the n-th harmonic amplitude is

$$|a_n| = \sum_k J_{n+k}(\beta) J_k(\beta) \left| \frac{\kappa_{\text{ex}} a_{\text{in}}}{i(-\Delta + k\Omega) + \kappa/2} \right|. \tag{13}$$

As we show in Eq. (10), the n-th harmonic amplitudes can be experimentally characterized by measuring the heterodyne beating note at the frequency $\omega_\mu - n\Omega$ (with a factor determined by the LO intensity). To reveal the accuracy of our heterodyne characterizations, we show in Fig. S8 the agreement of the measured spectrum of the first-order beating note with the theoretical result by equation (12), where Ω = 2.903 MHz and the RF power is -2.5 dBm (β = 1.29).

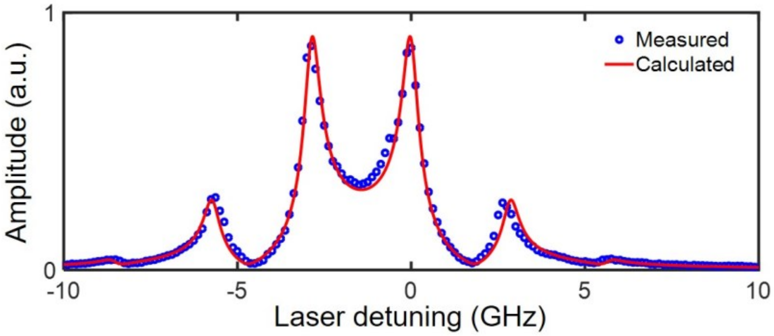

Fig. S8: Normalized optical spectrum of the first-order beating note obtained at Ω = 2.903 GHz and -2.5 dBm RF power (*β* = 1.29).

The characterization scheme also works for large modulation indices observed at Ω = 803 MHz. Within our HPD bandwidth (12 GHz), we show in Fig. S9 the examples of the fit to demonstrate that all the substantial harmonic signals can be read out with high fidelity (β = 6.90).

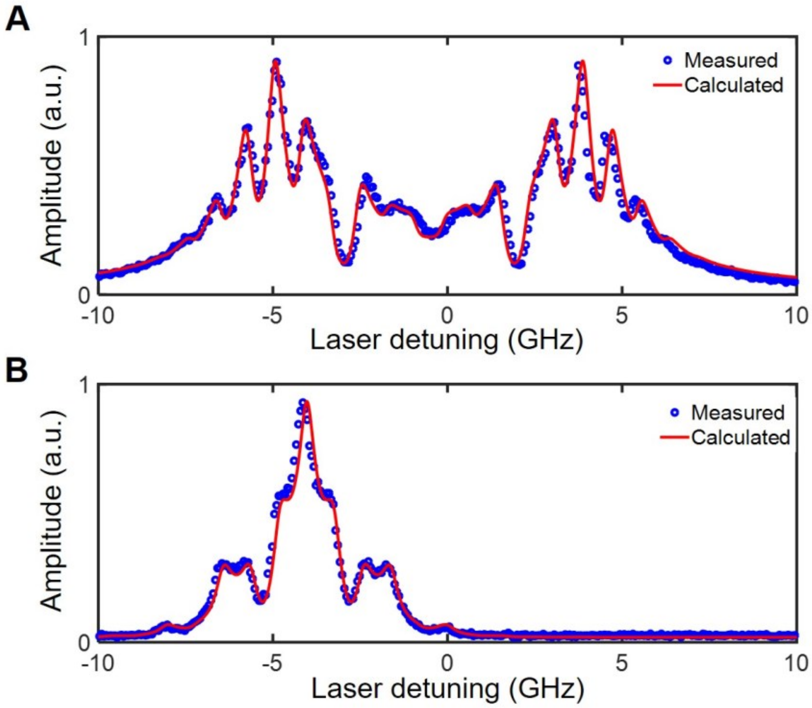

Fig. S9: Normalized optical spectra of the beating note obtained at Ω = 803 MHz and -17 dBm RF power (*β* = 6.90). (**A**) First-order beating note. (**B**) 10th-order beating note.

Supplementary Note 9. Driving phase dependence of the matrix-vector multiplications

The optomechanical coupling matrix represented by equation (3) in the main text has a dependence on the phase of the RF drive applied on the IDT. Fig. S10 displaces the theoretically calculated dependence on the modulation phase ϕ when the device is driven at 803 MHz and with β = 11.3. While this phase variation maintains the amplitudes of the site-to-site couplings ($|g_{mn}|$), a strong phase anisotropy in g_{mn} can be observed, which leads

to very different MVM outputs for spectrally coherent vector input. Specifically, for $\phi = 0$, the adjacent columns of **G** have minimum phase contrast, while the long-range coupling phases are considerable. Therefore, in our phase-coherent MVM demonstrations (Fig. 3 in the main text), we chose to set $\phi = 0$ so as to emphasize the persistence of the phase information of the long-range couplings.

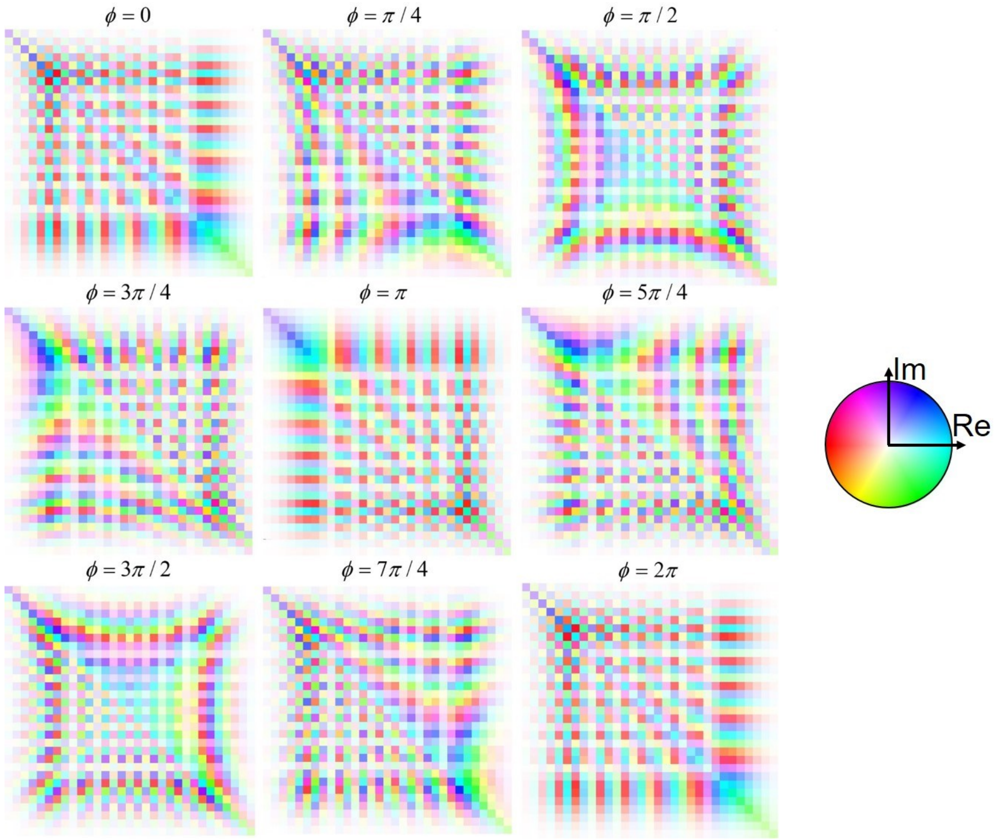

Fig. S10: Optomechanical coupling matrices at various microwave driving phase. The complex-valued matrix entries are represented by the false-color mapping. Matrix dimension is 30×30 at Ω = 803 MHz and β = 11.3.

<u>Supplementary Note 10. Measuring the output of the MVM operations on the synthetic frequency lattice</u>

The output amplitude at each frequency site, resulted from the MVM operations (Fig. 3 in the main text), was interrogated by the heterodyne measurements as we have described in Supplementary Note 5. The experimental setup for the large-scale MVM operations is shown in Fig. S11. The LO frequency $\omega_p + \omega_\mu$ is controlled by the tunable CW laser. When ω_p is set at one of the frequency sites ($\Delta = s\Omega$), the amplitude of the harmonic signal $|a_l|$ captured by the spectrum analyzer then corresponds to the amplitude at the synthetic lattice site of the order $s + l$. Therefore, we read out the output amplitudes at the synthetic frequency lattice by measuring all non-vanishing harmonic signals. We note, with a photodetector bandwidth of 12 GHz, we were able to fully interrogate up to 15th-order harmonic signals with high fidelity. The harmonic signals at even higher order are subject to decreased detection efficiencies, which set an upper bound for the size of the

synthetic lattice in our experimental demonstrations. By using a photodetector with higher bandwidth (> 40 GHz), our system can experimentally realize coherent 50×50 MVM operations with high-fidelity readout.

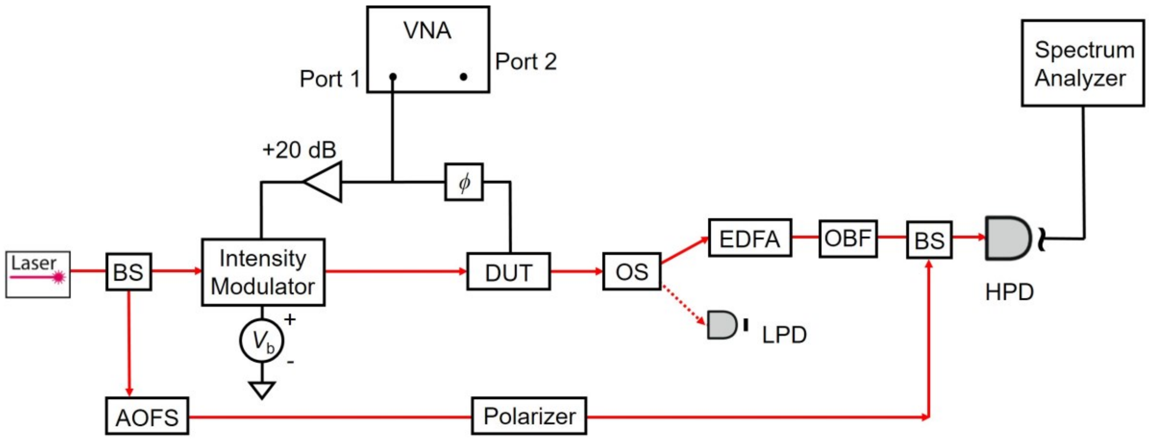

Fig. S11: Experimental setup of the large-scale MVM. The intensity modulator, which is driven by the VNA and a DC bias V_b, is cascaded before our device to provide a vector input of three spectrally coherent components. The RF phase shifter, ϕ, is used to tune the modulation phase difference between the intensity modulator and our device, which can be monitored by the transmittance spectrum measurements by the LPD. An RF amplifier of 20-dB gain is applied to drive the intensity modulator in order to obtain pronounced sideband input frequency components for our device.

Supplementary Note 11. Interrogating the noncommutativity of concatenated modulators

Fig. S12 shows the experimental setups in which we realized the concatenations of our nanophotonic cavity acousto-optic modulator (**G**) with a fiber-coupled broadband electro-optic phase modulator (**M**), in both **G·M** and **M·G** orders. We assume that the modulation phases of the RF drives **G** and **M** are ϕ_1 and ϕ_2, and the optical delay between the two modulators is τ. Under the same RF driving tone Ω, the modulation waveforms for the **M·G** order are

$$\begin{cases} \hat{f}_{\mathbf{M}}(t) = \cos(\Omega t + \phi_2) \\ \hat{f}_{\mathbf{G}}(t) = \cos[\Omega(t+\tau) + \phi_1] \end{cases}, \tag{14}$$

whereas for the reverse order (**G·M**), the modulation waveforms are

$$\begin{cases} \hat{f}_{\mathbf{M}}'(t) = \cos[\Omega(t+\tau) + \phi_2'] \\ \hat{f}_{\mathbf{G}}'(t) = \cos(\Omega t + \phi_1') \end{cases}. \tag{15}$$

We define the modulation phase differences $\Delta\phi = \arg\{\hat{f}_{\mathbf{G}}(t)\} - \arg\{\hat{f}_{\mathbf{M}}(t)\} = \Omega\tau + \phi_1 - \phi_2$ and $\Delta\phi' = \arg\{\hat{f}_{\mathbf{G}}'(t)\} - \arg\{\hat{f}_{\mathbf{M}}'(t)\} = \phi_1' - \phi_2' - \Omega\tau$. In our experiments, we controlled $\phi_1 - \phi_2$ ($\phi_1' - \phi_2'$) by the RF phase shifter with a tunable phase range [0, 2π]. We calibrated the optical phase delay τ to unify the two modulation phase differences associated with the two concatenation orders, by comparing the phase dependences of output amplitudes with the theoretical values.

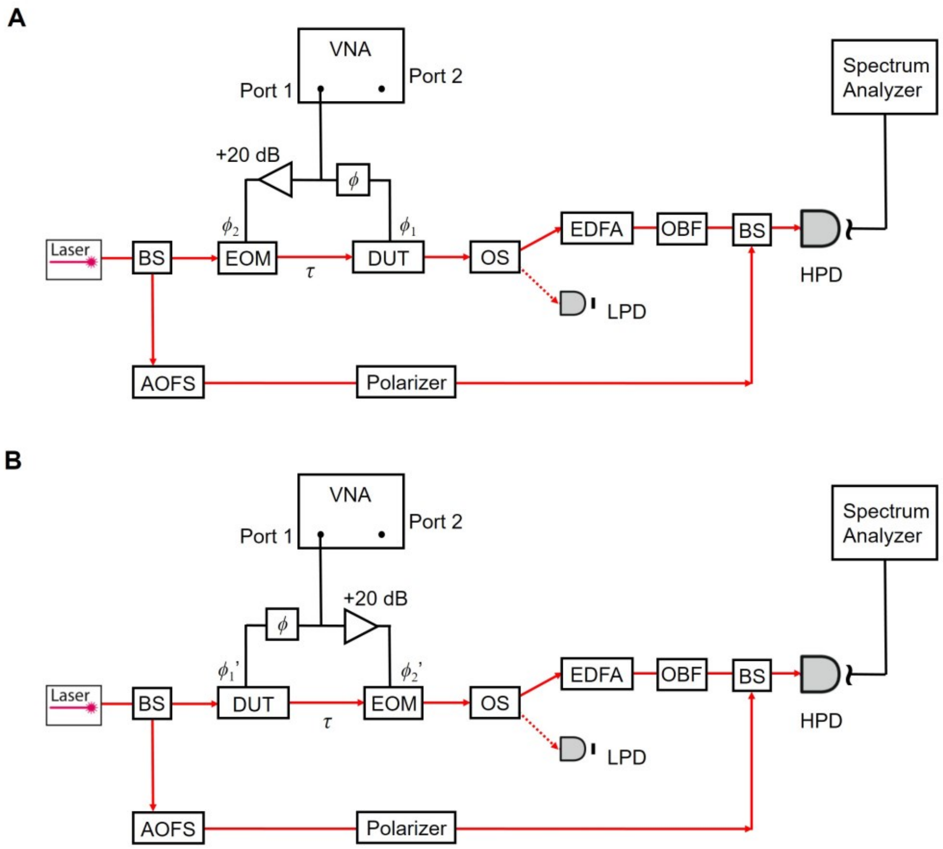

Fig. S12: Experimental setup of concatenated phase modulators. A broadband electro-optic phase modulator, driven by the VNA and a 20-dB RF amplifier, is cascaded before (**A**) and after (**B**) our device to realize the ordered matrix-matrix multiplications. The RF phase shifter (ϕ) is used to tune the modulation phase difference between the electro-optic phase modulator and our device. The fiber connection and fiber-chip coupling contribute to an optical delay line τ.

To understand the noncommutativity of the non-abelian group formed by cascaded phase modulators, we analyze the matrix-matrix multiplications between **M**: $[m_{kl}]$ and **G**: $[g_{kl}]$. The matrix of a broadband modulator is tri-diagonal represented by the carrier transmission on the diagonal entries m_{kk} and the two opposite-sign sideband generations $m_{k(k\text{-}1)}$ and $m_{k(k+1)}$ on the off-diagonal entries (ϕ_2 = 0). The matrix of our acousto-optic modulator is represented by equation (3) in the main text. For simplicity of the analytical calculations, we assume a moderate modulation index that only produces 1st order harmonics ($|k - l| < 2$). With a laser input at $\Delta = 0$, the resulted output vector through $\mathbf{y} = \mathbf{G} \cdot \mathbf{M} \cdot \mathbf{x}_0$ can be expressed as

$$\begin{aligned}
y_{-2} &= m_{-1,0}g_{-2,-1} \\
y_{-1} &= m_{0,0}g_{-1,0} + m_{-1,0}g_{-1,-1} \\
y_0 &= m_{-1,0}g_{0,-1} + m_{0,0}g_{0,0} + m_{1,0}g_{0,1} \; . \\
y_1 &= m_{0,0}g_{1,0} + m_{1,0}g_{1,1} \\
y_2 &= m_{1,0}g_{2,1}
\end{aligned} \tag{16}$$

For comparison, the output vector through $\mathbf{y}' = \mathbf{M} \cdot \mathbf{G} \cdot \mathbf{x}_0$ is

$$
\begin{aligned}
&y_{-2}' = m_{-2,-1} g_{-1,0} \\
&y_{-1}' = m_{-1,-1} g_{-1,0} + m_{-1,0} g_{0,0} \\
&y_0' = m_{0,-1} g_{-1,0} + m_{0,0} g_{0,0} + m_{0,1} g_{1,0} \, . \\
&y_1' = m_{1,1} g_{1,0} + m_{1,0} g_{0,0} \\
&y_2' = m_{2,1} g_{1,0}
\end{aligned} \tag{17}
$$

Using $m_{k(k+1)} = -m_{k(k-1)} = m$, $m_{l(l\pm1)} = m_{k(k\pm1)}$ and $m_{kk} = m_{ll}$, we obtain $\Delta\mathbf{y} = (\mathbf{G}\cdot\mathbf{M} - \mathbf{M}\cdot\mathbf{G}) \cdot \mathbf{x}_0$, where

$$
\begin{aligned}
&\Delta y_{-2} = m(g_{-2,-1} - g_{-1,0}) \\
&\Delta y_{-1} = m(g_{-1,-1} - g_{0,0}) \\
&\Delta y_0 = m(g_{0,-1} + g_{-1,0} - g_{1,0} - g_{0,1}) \, . \\
&\Delta y_1 = -m(g_{1,1} - g_{0,0}) \\
&\Delta y_2 = -m(g_{2,1} - g_{1,0})
\end{aligned} \tag{18}
$$

From here, we can attribute the arising of the noncommutativity to two aspects of the optomechanical coupling matrix of our modulator: 1) unlike the broadband EOM, the transmission through the modulated nanophotonic cavity at the center frequency $g_{0,0}$ is significantly different than that from the sidebands ($g_{1,1}$ and $g_{-1,-1}$), i.e. the non-unitarity, associated with the synthetic lattice of our resonating acousto-optic modulator, therefore $\Delta y_{\pm1}$ are generally nontrivial and are more pronounced at larger transmission difference; 2) as we have explained in Supplementary Note 9, the two-way frequency conversions between a pair of sidebands (g_{kl} and g_{lk}) are highly phase-anisotropic and have a strong dependence on the driving phase ϕ, which result in non-vanishing contrast at the center-frequency component (Δy_0) of the output. While our demonstrations involve a broadband and a resonating phase modulators, we note that these two factors ensure the sufficient and necessary conditions for the noncommutativity of the cascaded phase modulator group with the full parametric space of $(\beta, \Omega, \phi, \omega_0, \kappa, \kappa_{\text{ex}})$.

Supplementary Note 12. Enhancing programmability of the frequency-domain matrix-vector multiplication using modulation waveform shaping

A high degree of programmability in the matrix-vector multiplications is crucial for practical optical computation. In addition to cascading modulators, another approach that can enhance the programmability of the frequency-domain MVM in our device is to synthesize flexible periodic acousto-optic modulation waveform from harmonic acoustic wave activations.

To see how an arbitrary periodic acoustic waveform controls the optomechanical coupling matrix, we assume our device is modulated by an acoustic wave with a fundamental angular frequency Ω as

$$\hat{f}_{\text{wave}}(t + 2\pi/\Omega) = \hat{f}_{\text{wave}}(t) = \sum_{\nu} s_{\nu} \cos(\nu\Omega t + \phi_{\nu}) \, , \tag{19}$$

where s_ν, ϕ_ν are the normalized amplitude and phase of the harmonic tone at $\nu\Omega$, respectively. Under this modulation waveform, the intra-cavity photon dynamics reads

$$\dot{a}(t) = i\Delta a(t) - i\beta \cdot \Omega \cdot \sum_{\nu} \hat{s}_{\nu} \cos(\nu\Omega t + \phi_{\nu}) a(t) - \kappa a(t)/2 + \sqrt{\kappa_{\text{ex}}} a_{\text{in}}(t) \, , \tag{20}$$

where $\hat{s}_v = s_v \cdot v\beta_v / \beta$ is the modulation weight of the $v\Omega$ tone, and β_v is the corresponding modulation index at $v\Omega$ ($\beta_1 = \beta$). Similar to the solution in Supplementary Note 3, we use the transformation

$$a(t) = \alpha(t) \cdot \exp\left[-i\beta \sum_v \frac{\hat{s}_v}{v} \sin(v\Omega t + \phi_v) \right], \quad (21)$$

and rewrite equation (20) as

$$\dot{\alpha} = (i\Delta - \kappa/2)\alpha + \sqrt{\kappa_{\text{ex}}} e^{i\beta \sum_v \frac{\hat{s}_v}{v} \sin(v\Omega t + \phi_v)} a_{\text{in}} . \quad (22)$$

For simplicity of the analytical expression, we write the Fourier series of the Jacobi–Anger expansion as

$$\exp\left[i\beta \sum_v \frac{\hat{s}_v}{v} \sin(v\Omega t + \phi_v) \right] = \prod_v [\sum_k J_k(\hat{s}_v \beta / v) e^{ik(v\Omega + \phi_v)t}] \equiv \sum_k x_k(\{s_v\},\{\phi_v\}) e^{ik\Omega t} . \quad (23)$$

With $\alpha(t) = \sum_k \alpha_k \exp(ik\Omega t)$, we then arrive

$$ik\Omega \alpha_k = (i\Delta - \kappa/2)\alpha_k + x_k(\{s_v\},\{\phi_v\}) a_{\text{in}}, \quad (24)$$

and

$$\alpha_k = x_k(\{s_v\},\{\phi_v\}) \frac{a_{\text{in}} \sqrt{\kappa_{\text{ex}}}}{i(-\Delta + k\Omega) + \kappa/2} . \quad (25)$$

The intra-cavity photon dynamics and the output optical field are derived as, respectively,

$$\begin{aligned} a(t) &= e^{-i\beta \sum_v \frac{\hat{s}_v}{v} \sin(v\Omega t + \phi_v)} \cdot \sum_k \alpha_k \exp(ik\Omega t) \\ &= \sum_n e^{-in\Omega t} \sum_k x_{n+k}(\{s_v\},\{\phi_v\}) \cdot x_k(\{s_v\},\{\phi_v\}) \frac{\kappa_{\text{ex}} a_{\text{in}}}{i(-\Delta + k\Omega) + \kappa/2} \end{aligned}, \quad (26)$$

$$a_{\text{out}}(t) = \sum_n e^{-i(\omega_{\text{p}} + n\Omega)t} \sum_k x_{n+k}(\{s_v\},\{\phi_v\}) \cdot x_k(\{s_v\},\{\phi_v\}) \frac{\kappa_{\text{ex}} a_{\text{in}}}{i(-\Delta + k\Omega) + \kappa/2} . \quad (27)$$

The optomechanical coupling matrix corresponding to the modulation waveform is therefore

$$\mathbf{G} = [g_{m,n}] : g_{m,n} = \sum_k x_{m-n+k}(\{s_v\},\{\phi_v\}) \cdot x_k(\{s_v\},\{\phi_v\}) \frac{\kappa_{\text{ex}}}{i(-n+k)\Omega + \kappa/2} . \quad (28)$$

Changing of the periodic modulation waveform is equivalent to the control of the coefficients s_v and ϕ_v for each harmonic tone, which in turn alters the entries of the coupling matrix **G** in equation (28). It is therefore obvious that the MVM operations can be programmed by the modulation waveform shaping, where the number of independent variables increases as $2N$ for a synthesized waveform comprising N harmonic tones.

Experimentally, realizing more flexible modulation waveform in the acousto-optic modulator requires efficient actuation of the acoustic modes at multiple harmonic frequencies and accurate controls of their amplitudes and phases. To this end, many technical advances have been made to successfully generate multi-harmonic acoustic waves and synthesize arbitrary acoustic waveform by designs of the electromechanical transducer. For example, Ref. 38 and Ref. 39 (revised version) use frequency-chirped interdigital transducers (IDT) to generate up to $N = 5$ harmonic tones of acoustic modes. These IDT designs can be readily applied on our heterogeneous integrated platform to

enable the conversions of the RF drive into the desired acoustic tones. Additionally, the amplitudes and phases of the harmonic acoustic waves can also be programmed by the RF drive from an arbitrary waveform generator (AWG). Thus, future experimental work will be able to implement the modulation waveform programmability on our current AlN-on-SOI acousto-optic platform and thereby facilitate more hardware-efficient realizations towards arbitrary MVM operations in the synthetic frequency dimension.

Supplementary Note 13. Applications of frequency-domain MVM in an optical neural network

Benefiting from the synthetic frequency dimension (sideband space), our nanophotonic cavity acousto-optic modulator fulfills large-scale linear complex-valued matrix-vector multiplications (MVM) in a hardware-efficient way, thus promises the advance of optical computing systems such as optical neural networks. To demonstrate such a prospect, here we design and simulate a multilayer perceptron neural network for image recognition and classification, where the computationally intensive linear MVM operations are performed in the synthetic frequency dimension using our devices (Fig. S13). Pixelated images are first color-mapped into rows of complex-valued vectors and encoded on the basis of the optical sidebands. The fully-connected linear MVM layer is physically implemented by an array of modulators connected in parallel (beam-splitting) and/or series (cascading) (lower inset, Fig. S13A), which compose the total matrix operator in the synthetic frequency dimension

$$\mathbf{G} = \sum_q w_q \left(\prod_p \mathbf{G}_q^p\right),$$

where w_q are the weights of the modulator series branches controlled by the beam splitting, $\mathbf{G}_q^p$ is the coupling matrix of the indexed modulator, p and q are the indices of the modulators in series and in parallel, respectively. The vectors after the MVM are subsequently activated by a nonlinear function $F(x)$, and sent to a pooling layer which yields the output of the network.

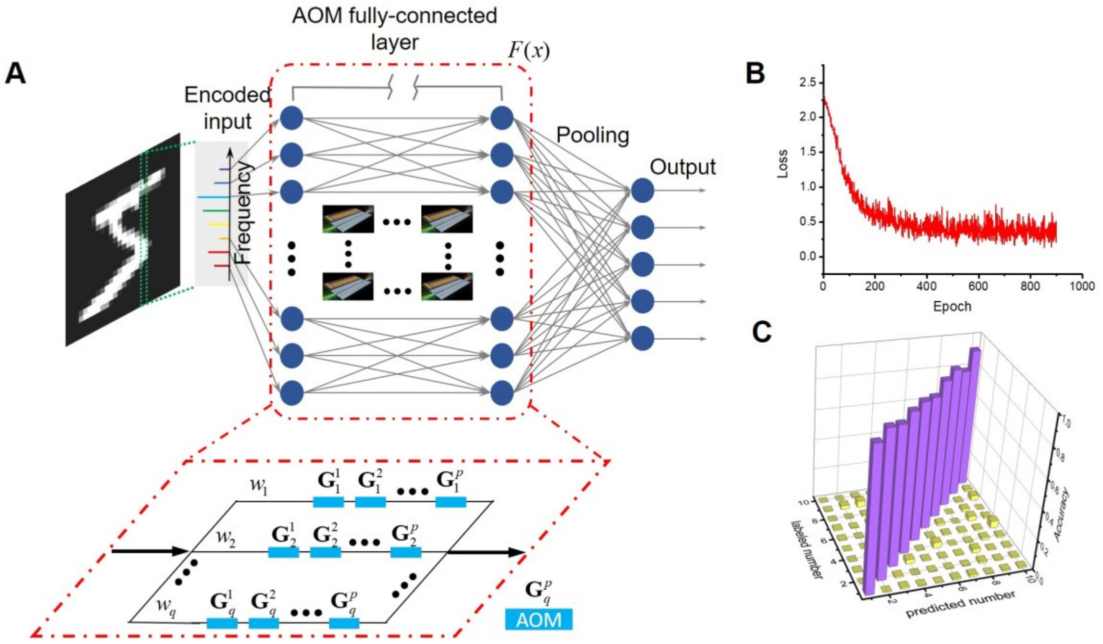

Fig. S13: Simulations of an optical multilayer perceptron neural network that incorporates frequency-domain MVM. (**A**) Neural network model that can recognize and classify hand-written digits. (**B**) Typical loss function evolution as the training progresses. (**C**) Digit recognition accuracy at the completion of the training.

In our simulations, we test the network for recognition of handwritten digits using the standard MNIST database. The 28×28, 8-bit grayscale images of handwritten digits are encoded in 28 vectors of size 28×1, and are fed into the input layer column by column. To prove the concept, we design the MVM layer by three copies of our acousto-optic modulators in parallel ($p = 1$, $q = 1,2,3$), and split each input vector into three paths with the weights w_1, w_2, w_3. The modulation index for each modulator is set around 10 to realize the 28×28 fully connected layer. The results of the MVM are read out, applied to a nonlinear activation function (sigmoid) and to the pooling layers. The output vectors of the network are compared with the prediction which defines the loss function. During the training process, we optimize the beam-splitting weights (w_1, w_2, w_3), modulation indices and phases of the three parallel modulators and the pooling layers, such that the loss function is minimized. Fig. S12B shows the decreasing of the loss function as the training process evolves with epochs. We reiterate the process for all digits from "0" to "9" and reach the accuracy over 90% in the simulations (Fig. S12C). It is worth noting that the 28×28 matrix operator that has enough trainability to carry out image recognitions in our simulations is realized by only three copies of our device. Our simulations therefore prove the merit of our device applications in optical computing.
